# Molecular, genetic and stem cell-mediated therapeutic strategies for spinal muscular atrophy (SMA)

**DOI:** 10.1111/jcmm.12224

**Published:** 2014-01-08

**Authors:** Chiara Zanetta, Giulietta Riboldi, Monica Nizzardo, Chiara Simone, Irene Faravelli, Nereo Bresolin, Giacomo P Comi, Stefania Corti

**Affiliations:** a Dino Ferrari Centre, Neuroscience Section, Department of Pathophysiology and Transplantation (DEPT), University of Milan, Neurology Unit, IRCCS Foundation Ca' Granda Ospedale Maggiore PoliclinicoMilan, Italy

**Keywords:** spinal muscular atrophy, molecular therapy, antisense oligonucleotides, morpholino, gene therapy, stem-cell therapy, induced pluripotent stem cells

## Abstract

Spinal muscular atrophy (SMA) is an autosomal recessive motor neuron disease. It is the first genetic cause of infant mortality. It is caused by mutations in the survival motor neuron 1 (*SMN1*) gene, leading to the reduction of SMN protein. The most striking component is the loss of alpha motor neurons in the ventral horn of the spinal cord, resulting in progressive paralysis and eventually premature death. There is no current treatment other than supportive care, although the past decade has seen a striking advancement in understanding of both SMA genetics and molecular mechanisms. A variety of disease modifying interventions are rapidly bridging the translational gap from the laboratory to clinical trials. In this review, we would like to outline the most interesting therapeutic strategies that are currently developing, which are represented by molecular, gene and stem cell-mediated approaches for the treatment of SMA.

- Introduction
- Molecular therapy
  —Antisense oligonucleotides
- Gene therapy
- Stem-cell therapy
- Conclusions

## Introduction

Spinal muscular atrophies (SMAs) are a group of hereditary autosomal recessive neuromuscular diseases characterized by degeneration of alpha motor neurons in the spinal cord and brainstem, resulting in progressive proximal muscle weakness, hyposthenia and paralysis, which are usually symmetrical. Spinal muscular atrophy is the leading genetic cause of infant mortality with an estimated incidence of 1 in 6000 to 1 in 10,000 live births, with a carrier frequency of 1/40-1/60 1, 2.

The classical form of the disorder is caused by genetic mutations 3 in 5q11.2–q13.3, affecting the survival motor neuron (*SMN*) gene 4 and leading to the absence of *SMN1* exon 7. Spinal muscular atrophy is conventionally clinically classified into four subtypes (SMA I, SMAII, SMAIII, SMA IV) on the basis of age of onset and highest motor function achieved with an additional phenotype (type 0) to describe the severe forms with an antenatal-onset 5. Prognosis depends on the phenotypic severity ranging from high mortality within the first year for SMA type I to no mortality for the chronic and later onset forms. The major modulator of clinical phenotype is the human paralogue gene *SMN2* that essentially differs from *SMN1* by 2 single nucleotide polymorphisms (SNPs) in exon7 and exon8 and by several SNPs in introns. Although no amino acid substitution is induced by the exonic SNPs, the substitution of a C with a T in exon 7 alters a splicing modulator, resulting in exon 7 exclusion in 90% of *SMN2* mRNA transcripts. The increased attention to early diagnosis and to several aspects of management of SMA has stimulated the development of clinical guidelines and standards of care 6, 7. Consequently, therapeutics development in SMA now has strong academic, government and industry involvement. The aim of this review was to give a general overview on the molecular, genetic and stem-cell therapeutic strategies for SMA that are currently ongoing.

## Molecular therapy

### Antisense oligonucleotides

**Fig 1:**
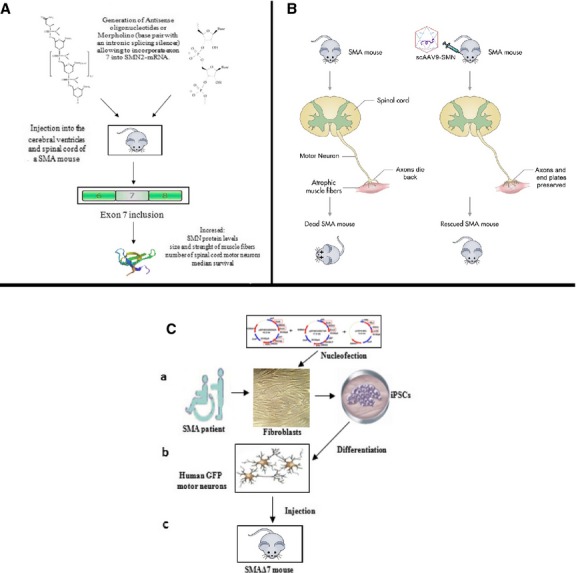
(A) Molecular therapeutical approach for spinal muscular atrophy (SMA): Antisense oligonucleotide or Morpholino binding to specific cis-acting splicing regulatory motifs can promote exon 7 inclusion and therefore increase the production of full length survival motor neuron (SMN) protein. (B) Gene-therapy approach: scAAV9-SMN delivery through intramuscular injection, systemic delivery or direct delivery to the central nervous system was demonstrated to improve SMA phenotype after early postnatal delivery in severe SMA mice models. (C) Stem cell-based therapeutic approach 49: (*a*) Generation of iPSC lines from two type I SMA patients using a non-viral vector method based on nucleofection of adult fibroblasts with constructs encoding OCT4, SOX2, NANOG, LIN28, c-Myc, and KLF4. These plasmids are progressively lost from cells. (*b*) Uncorrected SMA-iPSC-derived motor neurons reproduced disease-specific features, which were ameliorated in motor neurons derived from genetically corrected SMA-iPSCs. (*c*) Upon direct transplantation into a severe SMA mouse model, corrected SMA-iPSC-derived motor neurons engrafted in the spinal cord.

Recent evidence demonstrates the promising therapeutic potential of modulating *SMN2* splicing through molecular strategies including antisense oligonucleotides (ASOs), that are modified nucleotides that bind specific mRNA sequences (Table 1, Fig. 1A). This binding can mark a specific mRNA for degradation or, in the case of its use in SMA therapy, binding to specific cis-acting splicing regulatory motifs can promote exon 7 inclusion in *SMN2*. Antisense oligonucleotides need to highly promote exon retention, be resistant to cellular degradation and have high target specificity, low toxicity and high penetrance to target cells including neurons within the central nervous system (CNS). Also, ASOs can be chemically modified to become resistant to cellular endonuclease activity and operate independently of RNase-H degradation. Spinal muscular atrophy represents an incredibly suitable target for the use of ASOs because of the presence of the defective *SMN2* rescue gene, the duality of both weak 3′ and 5′ exon 7 splice-sites and the presence of many local and regional splicing silencers and enhancers. Various ASOs with different chemistries 8–10 have been studied in cell and animal models, and many molecules have been designed to target an intronic splicing silencer (ISS) sequence in the 5′ end of intron 7 11–13. The three main different chemistries of ASOs are: 2′-O-methyl phosphorothioate (2OMePS), 2′-O-Methoxyethyl (MOE), and morpholino (PMO). Bifunctional ASO sequences targeting exon 7 or nearby can efficiently use the great amount of splice activators and suppressors by combining a sequence specific ASO with a tethered oligomer that works as a binding platform for positive splice activators. In literature, different ASO chemistries have been used (2OMePS, MOE and peptide nucleic acid) and many researchers showed this proof of concept by targeting the putative exonic splicing enhancer abolished by C6T transition, combined with a tailed splice-factor recruiting ASO 14, 15, with the result of increasing splicing to *SMN1* levels. Moreover, a similar 3′ ss exon 7 targeting scheme that used lentiviral germ line transgenesis was also used to increase exon 7 retention and SMN protein levels 16. In literature, two studies in which bifunctional ASOs targeting splice suppressor sequences have been combined with tethered splice activator recruiters of SR-like proteins (SF2/ASF, hTra2β1) are available 17, 18. Osman *et al*. were able to sterically block the ISS-N1 sequence with a 2OMePS chemistry ASO intracerebroventricularly (ICV) delivered three times before postnatal day 5 (P5), with the result of increasing SMN protein up to 3.5-fold within the CNS, although the median survival was modestly increased in the SMAΔ7 mouse model (median survival increased to 19–20 days, which is 46.1–53.8% increase) 17. Baughan *et al*. blocked the E1 splice suppressor upstream of exon 7 with ICV injections of a 2OMePS chemistry ASO. Survival motor neuron protein throughout the neuraxis increased up to twofold, although delayed analysis at 5 days showed little or no effect. Moreover, severe SMA mice (Smn−/−; SMN2+/+) had a very modest increase in the survival (16.7%) and bw (33.3% increase from post natal day 2 to post natal day 5) and this could be for many reasons: low ASO doses, multiple cerebral cannulations, delayed initial treatment and potential CNS toxicity from this chemistry 18. Other strategies have targeted ASOs to the intron 7/exon 8 junction with the goal of reducing recognition of the exon 8 3′ ss 19, 20. The most important splice modulator that has been so far targeted by ASOs for SMA is the ISS number 1 (ISS-N1) sequence adjacent to the exon 7 5′ ss. Antisense oligonucleotide microwalks are able to strongly alter splicing when targeting intron positions 10-27, 10-29, 10-34 and 07-14 13, 21–24. There have been many studies on ASO ISS-N1 therapy. One of them demonstrated that an ICV injection of a relatively low dose (10 μg through 10 ICV cannulations) of a 20-nucleotide 2OMePS into SMAΔ7 mice leads to a full-length SMN increase in the CNS and improves righting [at three levels of stringency (≥1/6, ≥2/6, ≥3/6), respectively, 30%, 20% and 20% more of the treated mice were able to right themselves at P12 compared with untreated and scramble-treated mice] and weight gain (66.7% increase in treated mice compared to 33.3% increase in untreated and scramble-treated mice), even though survival was not evaluated 8. Dose elevation analysis of an 18-nucleotide MOE sequence into a mild mouse model showed that continuous CNS infusion into adult animals of up to 100 μg/day were well tolerated and produced effective and long-lasting splice modulation 9. Embryonic and neonatal ICV bolus therapy (20 μg maximum dose) delayed necrosis onset. In a direct comparison of 2OMePS and MOE chemistries, the former showed increased spinal cord inflammatory markers as well as reduced capacity for exon 7 inclusion. Further investigation of the MOE chemistry after ICV bolus delivery in the neonatal SMNΔ7 mouse showed strong exon 7 retention throughout the entire neuraxis, improvement in weight gain (on average, >50% increase in the treated mice at 16 days), motor function (at 16 days, 420% increase in force in the treated mice), increased motor neuron count (57.1%, 50% and 7% increase in the cervical, thoracic and lumbar regions of treated mice, respectively, compared to SMNΔ7 mice) and neuromuscular junction architecture (quadriceps: at 16 days, ∼92% of neuromuscular junctions collapsed in the SMNΔ7 mice, whereas only 13% in the treated mice. Intercostal: at 16 days, 85% of neuromuscular junctions collapsed in the SMNΔ7 mice, whereas only 12% in the treated mice), while median survival was only modestly extended (26 days, 100% increase) 10. Hua *et al*. 12 found a slightly increased survival after a 20 μg bolus MOE ICV delivered. Strikingly, multiple subcutaneous (SC) or intraperitoneal (IP) injections of large dose MOE (320 μg ASO/g) resulted in 248-day median survival (1553.4% increase), with some animals living >500 days (3233.4% increase). Peripheral tissues, in particular the liver, showed large increases in SMN protein; brain and spinal cord also showed a fourfold increase in exon 7 retention, suggesting ASO crossing of the immature blood–brain barrier (BBB) of the neonatal mouse. Although this study pointed out the importance of SMN peripheral rescue, it is important to consider that even a modest elevation of SMN within the CNS can affect survival 25–27. Recently, it has been demonstrated by Singh *et al*. the existence of a new target sequence, named ISS-N2, which is able to increase intron 7 inclusion in *SMN2* when targeted by ASOs 29. The discovery of this new sequence opens the way to new molecular targets that can be tested in preclinical study and subsequently in clinical trial.

**Table 1:** Molecular, Genetic and Cellular transplantation approach to treat SMN deficiency. When there were more tests for a single compound with different dosages we reported just the experiment with a greater survival

	Strategy	Outcome	References
Molecular therapy			
*Antisense oligonucleotides*	Compound: ASO towards the 3′ splice site of exon 8 of *SMN2 Model: G418 cell line*Route of administration:-	Increased exon7 inclusion in G418 cells transfected with SMN1 and 2 mini-gense	Lim *et al*. 19
	Compound: anti-SMN U7 snRNAsModel: HeLa cell lineRoute of administration:-	40% increased SMN expression in HeLa cells expressing endogenous *SMN1* and *SMN2*	Madocsai *et al*. 20
	Compound: 2′-O-methyl ASO against ISS-N1Model: SMA fibroblast line GM03813Route of administration:-	Increased SMN expression	Singh *et al*. 11
	Compound: ASO 07–21, ASO 34–48 Model: SMA type I 3813 fibroblastsRoute of administration:-	Increased SMN protein level: 1.8-fold with ASO 07–21 and 2.1-fold with ASO 34–48	Hua *et al*. 12
	Compound: Bifunctional U7 snRNA (antisense U7 snRNAs with an ESE)Model: SMA fibroblast lineRoute of administration:-	2-to 2.7-fold increased SMN protein amount after transduction with lentiviral vectors of human fibroblasts from a SMA type I patient	Marquis *et al*. 16
	Compound: ASO 10–27, ASO 09–23 Model:human *SMN2* transgenic mice	Increased exon 7 inclusion in mouse liver and kidney, but not in spinal cord	Hua *et al*. 13
	Route of administration: systemic		
	Compound: Tra2-E1 (2′-O-methyl bifunctional RNA)Model: SMA fibroblast line 3813, SMNΔ7 miceRoute of administration:-, ICV	Increased SMN expression in SMA patient 3813 fibroblasts and in SMNΔ7 mice after ICV injection; increased lifespan and weight gain (∼30%) in the SMN2+/+Smn−/− mouse	Baughan *et al*. 18
	Compound: 2OMePS 8-mer ASO binding upstream of ISS-N1Model: GM03813 SMA cell lineRoute of administration:-	Significant up-regulation of SMN levels in GM03813 (SMA type I patient cells)	Singh *et al*. 23
	Compound: 2′-O-methyl ASOModel: SMNΔ7 miceRoute of administration: ICV	Increased SMN expression in CNS, motor performance and weight in SMA mice	Williams *et al*. 8
	Compound: ASO 10-27Model: type III SMA miceRoute of administration: systemic	Delayed tail and ear necrosis	Hua *et al*. 9
	Compound: ASO 10-27 (2′-O-(2-methoxyethyl phosphorothioate-modified ASO))Model: SMNΔ7 mice (average lifespan=15 days)Route of administration: ICV	The median lifespans of ASO 10-27–treated SMA mice were 23 (53.3% increase; 8 μg, n = 20), 25 (66.7% increase; 4 μg, n = 29), 23 (53.4% increase; 2 μg, n = 12), 20 (33.4% increase; 1 μg, n = 13), and 17 days (13.4% increase; 0.5 μg, n = 13), compared to 16 days (6.7% increase) for both the ASO-mismatch–treated (4.0 μg, n = 19) and untreated (n = 30) SMA mice	Passini *et al*. 10
	Compound: Bifunctional ISS-N1 ASO Model: SMNΔ7 mice (average life span=15 days)Route of administration: ICV	Prolonged survival to 19-20 days (33.3% increase)	Osman *et al*. 17
*Antisense Oligonucleotide Morpholino*	Compound: MO HSMN2Ex7D(-10-29) against ISS-N1Model: SMNΔ7 mice (average life span=15 days)Route of administration: ICV	Median survival 112 ± 6.6 days (646.7% increase) with high dose (81 μg)	Porensky *et al*. 22
	Compound: PMO(-10-29), PMO(-10-31), PMO(-10-34) complementary to ISS-N1 Model: (mSmn−/−; SMN2+/+; Δ7+/+) mice (average life span=15 days)Route of administration: ICV	Median survival (6 mM):-PMO(-10-34): 135.3 (802% increase) days-PMO(-10-29): 126 (740% increase) days	Mitrpant *et al*. 21
	Compound: PMO25 (-10-34), PMO18 (-10-27) and PMO20 (-10-29) complementary to ISS-N1-VMO25Model: SMA mice.-severe (average lifespan= 9.5 days) and mild SMA mice (average lifespan unknown)Route of administration: ICV or IV-IV and IP	ICV:-PMO25: 185 days (1847.3% increase; 20 μg), 285 days (2900% increase; 40 μg);-PMO18: 25 days (163.1% increase; 20 μg), 32 days (236.8% increase; 40 μg)VMO25:-severe SMA mice: 16 days (68.4% increase)-mild SMA mice: delayed tail and ear necrosis	Zhou *et al*. 24
Gene therapy	Compound: EIAV-SMNModel: SMA mice (average life span= 13 days)Route of administration: intramuscular	Average survival: 18 ± 1.5 days (38.5% increase)	Azzouz *et al*. 41
	Compound: scAAV9-SMNModel: SMAΔ7 mice (average lifespan= 15 days)Route of administration: systemic	More than 250 days (more than 1512.9% increase)	Foust *et al*. 32
	Compound: AAV8-hSMN and scAAV8-hSMN intoModel: SMA mice (SMN^−/−^, hSMN2^+/+^, SMNΔ7^+/+^; average life span= 15 days)Route of administration: direct administration in the CNS	AAV8: median survival 50 days (233.4% increase); scAAV8-hSMN: median survival 157 days (946.7% increase)	Passini *et al*. 33
	Compound: scAAV9-coSMNModel: SMNΔ7 mice (average lifespan= 15 days)Route of administration: IV	Average survival: 69 days (360% increase), maximum survival: 190 days (1166.7% increase)	Valori *et al*. 34
	Compound: scAAV9+ codon-optimized hSMN1Model: mouse model of severe SMA (average lifespan= 15 days)Route of administration: IV and intraspinal	Median survival: 199 days (1226.7% increase), longest survival: 340 days (2166.7% increase)	Dominguez *et al*. 35
	Compound: scAAV9-SMNModel: SMNΔ7 mice (average lifespan= 15 days)Route of administration: ICV and IV	Average survival: ICV 17 days (13.3% increase), IV 10 days	Glascock *et al*. 44
Stem-cell therapy	Compound:ALDHhiSSClo neural stem cellsModel: B6.BKS Ighmbp2nmd^−2J^ mice (average life span= 3.5–4 weeks)Route of administration: intrathecally	Mean survival: males 74.9 ± 13.3 days; females 88.3 ± 17.1 days; longest survival: male 107 (256.7% increase), female 127 days (323.4% increase)	Corti *et al*. 47
	Compound:NSCModel: SMNΔ7 mice (average lifespan= 15 days)Route of administration: intrathecally	Survival of 18.16 ± 1.78 days (∼21% increase)	Corti *et al*. 48
	Compound:Human embryonic stem cell-derived motor neuron progenitors (hMNPs)Model: SMAΔ7 mice (average lifespan= 15 days)Route of administration: intrathecally	Cell engraftment and differentiation in the anterior horn of SMAΔ7 mice spinal cord with increased sparing of spinal cord neurons caudal to the injection site	Wyatt *et al*. 50
Compound:iPSC with genome editing of SMN2Model: SMAΔ7 mice (average lifespan= 15 days)Route of administration: intrathecally	Increased lifespan of SMAΔ7 mice up to 21 days (40% increase)	Corti *et al*. 51	

CNS: central nervous system; ICV: intra cerebro ventricular; IV intra venous; iPSC: induced pluripotent stem cell, NSC: neural derived stem cell; AAV: adeno-associated virus; scAAV: self-complementary adeno-associated virus; hSMN: human survival motor neuron; EIAV: Equine infectious anaemia virus; IP intra peritoneal; VMO: vivo morpholino (conjugated with a dendrimeric octaguanidine); ASO: antisense oligonucleotide; SC: subcutaneous; ESE: exonic splicing enhancer.

Antisense oligonucleotide therapy can drastically increase survival in SMA models and should proceed forward as a potential strong disease modifier. For this reason, early clinical trials are ongoing and others are starting to assess safety and pharmacokinetics of single dose MOE ASO delivered by lumbar intrathecal injections in children with SMA (ISIS Pharmaceuticals http://clinicaltrials.gov/). We already know from clinical studies in other neuromuscular diseases, such as Duchenne Muscular Dystrophy (DMD), that molecular therapy with ASOs has different adverse events, such as cutaneous irritation and renal sufferance 29. Data of safety and efficacy after ASO therapy administration in children affected by SMA are not available up to now, but these pioneering clinical studies will, for sure, contribute to define the best dosage to reduce adverse events and reach positive therapeutic effects.

#### Morpholino

MO is an ASO in which the phosphorothioate-ribose backbone is replaced by phosphorodiamidate-linked morpholine backbone. This modification leads to an increased resistance to metabolic degradation. MO is characterized by low toxicity and it is already in use for clinical trials for DMD 30. Antisense oligonucleotide-induced interference with splicing will likely be one of the first molecular therapies for SMA that will approach the clinics (Table 1). In particular, for reasons of its excellent stability, safety and efficacy profile, MO will be one of the most promising candidates. However, some critical issues are still unresolved, such as the optimal type of MO chemistry and sequence and the modalities of administration. It is not obvious if local injection is sufficient to rescue SMA phenotype 22 or if systemic injection is necessary and sufficient 31. As described by Mitrpant *et al.,* increasing the oligonucleotide length can enhance efficiency of ASO to promote full-length SMN. Fourteen different PMOs varying in size of 20, 22 and 25 nucleotides were designed against or near ISSN1 (-10-25) and were transfected into fibroblasts derived from a SMA Type I patient 21. Taking into account the five PMO sequences of 20 nucleotides directed between intronic bases 8 and 35 downstream of exon 7, PMO(-10-29) was the most efficient in inducing full-length *SMN2*. When the length of PMO(-10-29) was extended by five bases at the 5′ end, PMO(-10-34), a further improvement in the efficiency of exon inclusion in *SMN2* was reached. In contrast, extending PMO length by 5 bases at the 3′end, PMO(-5-29) led to the reduction in *SMN2* exon 7 inclusion levels. Similarly, a 25 nucleotide PMO sequence that targeted intron 7 regions further downstream of ISS-N1 (-14-38) was not able to promote exon 7 inclusion. After trying to use PMO sequences with 22 nucleotides, it has been further demonstrated that the window for PMO targeting is very narrow. PMO(-10-31) generated an obvious dose-dependent exon 7 retention, whereas PMO(-12-33) was essentially inactive, as were other 22 nucleotides PMO (PMO(-1435) and PMO(-16-37)) 21. Mitrpant *et al*. undertook titration studies of the three lead PMO candidates (20, 22 and 25mers) to demonstrate dose responses and identify the lead compound. RT-PCR showed that SMN exon 7 inclusion was induced by PMO(-10-34) at low dose, while the 20 and 22mers PMOs were active only when transfected at higher concentrations in a type I SMA fibroblast. PMO(-10-34) was selected for further *in vivo* evaluation. 21. These PMOs and scrambled PMO were administered into SMA mice. After a single ICV dose of PMO into SMA mice, survival was maximum with PMO(-10-34), compared to PMO(-10-29) ranging from 15 days to a median range of 37 (146.7% increase) to 126 days (740% increase), depending on the tested dose 21. The weight gain was coherent with survival data. Overall, the optimal PMO sequence is usually ∼25 nucleotides long. Also, Zhou and colleagues showed in a different mouse model that a PMO sequence of 25 nucleotides is superior to other previously described ASOs/PMO sequences 24.

In the perspective of clinical trials, both the best molecule and route of administration need to be elucidated. Therefore, it will be important to define whether a local CNS strategy *versus* a combined CNS/peripheral strategy will be more favourable in larger animals and in clinical trials. A very strong point of ASO-mediated therapy is that, in our opinion, this strategy seems to be appropriate for all SMA patients. Our belief is supported by the fact that the alteration in RNA splicing is a consequence of the genetic mutations that cause SMA, being present in patients affected by all forms of SMA, from the milder types to the most severe ones, and that ASOs are able to directly correct this alteration and therefore can be useful for all SMA patients who have a common disease pathogenesis. A question that still needs to be addressed is whether this treatment could be helpful for patients who already have an established disease, as a diagnosis of SMA before the development of the symptoms is not always easy and possible in patients. What seems to be obvious from a systematic analysis of the literature is that a treatment planned for the earliest stages of the disease leads to the best results in terms of survival and rescue of the phenotype. In particular, to study the effects of ASOs at a later stage of the disease, symptomatic SMA mice at post natal day 5–7 have been used by our group and other researchers finding a reduced restoration of the phenotype and a diminished increase in the survival, supporting the idea that early administration (post natal day 0 and 3) is more efficient. This could be for many reasons. First of all the blood–brain barrier(BBB) of small pups seems to be more permeable than the one of grown mice, because of the immaturity and ‘leakiness’ of the BBB of neonatal mice. This issue could be partially overcome by modifying the chemical structure of ASOs or through their conjugation with peptides and/or molecules that facilitate the penetration of cells and tissues. Nevertheless, a late MO administration could still not be as efficient as an early one because proper SMN protein levels could be fundamental for the correct development of SMA pups at an early stage of their lives, therefore leading to the fact that a late restoration of SMN levels could not be sufficient to rescue the phenotype. Taken together, these results support the idea that a therapeutic window to increase SMN protein expression exists. This aspect will be analysed in detail in the conclusions of this manuscript.

Overall, ASO-based therapy for the treatment of SMA seems to have an excellent therapeutic potential in SMA mice and warrants further development for rapid and successful translation into humans.

## Gene therapy

The main gene therapy approach for SMA currently in use focuses on the replacement of *SMN1* (Table 1, Fig. 1B). Gene therapy gives the opportunity to restore a normal form of *SMN1*; however, effective delivery to a difficult-to-access cell such as a motor neuron has been considered an almost impossible challenge until very recently when this difficulty has been overcome by using self-complementary adeno-associated virus vectors 32–35. scAAV9 was shown to have tropism for motor neurons in neonatal animals when injected intravascularly 36. Both SMN1 scAAV9 and scAAV8 were demonstrated to improve SMA phenotype after early postnatal delivery in severe SMA mice models. There are numerous viral vectors that have been shown to have a tropism for cells of the CNS, such as lentiviral vectors (LV) and adeno-associated viral vectors (AAV) 37–39. These vectors have the ability to transduce non-dividing cells and also give stable gene expression. The most direct approach is gene replacement with full-length SMN to increase levels of SMN protein. Three main routes of administration have been examined: intramuscular administration of a viral vector capable of retrograde transport to the motor neurons of the CNS, systemic delivery using a vector, which can cross the BBB or direct delivery to the CNS either *via* direct injection into the spinal cord or into the cerebrospinal fluid (CSF) *via* the intraventricular or intrathecal spaces. The first noteworthy gene therapy approach by Azzouz *et al*. was performed through intramuscular injections 41. Researchers chose an LV-based vector system for its ability to transduce non-dividing cells. The equine infectious anaemia virus (EIAV) vector was chosen over classic HIV-1-based LV system. The EIAV vector was pseudotyped with the glycoprotein for rabies virus (Rabies G), conferring the ability to be retrogradely transported 40. One of the advantages of using a vector capable of retrograde transport is the fact that it is a less invasive approach than direct injection into the spinal cord. When injected into the gastrocnemius, facial, intercostal or tongue muscle of postnatal day 2 (P2) mice, EIAV lac-Z gives robust expression of motor neurons in the spinal cord and brain stem respectively 41. There were increased levels of SMN protein in motor neurons with a limited increase in survival of Δ7 SMA model by up to 38% 41. The discovery that AAV9 vector is capable of crossing the blood–brain barrier opened up a new potential vector. Separate groups have shown that intravenous delivery of self-complementary AAV9 (scAAV9) can rescue the phenotype of SMA mice 32, 34, 35. Foust *et al*. delivered scAAV9 *via* the facial vein of P1 SMAΔ7 mice 32. Increased levels of SMN protein were observed, although it was still lower than that of control animals. scAAV9 SMN-treated animals showed improvement in motor testing (∼90% improvement in the righting time: 30 sec. for untreated *versus* 3 sec. for treated) when compared with untreated animals and control animals. The most important result of this work was the dramatic improvement in survival in scaAAV9SMN-treated animals that was more than 250 days (∼1566.7% increase). Two other groups have shown similar results with scAAV9SMN in Δ7 mice 34, 35. Both groups showed high levels of SMN protein expression with a 10-fold increase of SMN protein from the codon-optimized *SMN1* 34, 35. Dominguez *et al*. reported increased survival in all treated animals after a single injection into the temporal vein of P1 pups with scAAV9 codon-optimized *SMN1* (∼1130% increase) 35. Pre-clinical work has shown that AAV9 can lead to an increased motor neuron expression after delivery to the CSF 42. This is promising for the translation of AAV9 to the clinic, with regard to efficacy of transduction, feasibility of the route of administration and dose ranging. Direct injection of AAV8SMN into both the spinal cord and cerebral lateral ventricles of newborn Δ7 pups resulted in increased SMN protein expression 33. SMN expression on all levels of the spinal cord and in spinal cord motor neurons was observed, suggesting that the SMN protein is being expressed in the appropriate areas to have a therapeutic effect. Treated animals showed improved skeletal muscle size, neuromuscular junction structure and motor function. AAV8SMN was shown to increase survival to an average of 50 days, correlating with 233% increase. However, this increase in survival is modest when compared with the rescue provided by scAAV9 or scAAV8 (250 and 157 days, respectively, which correspond to 1566.7% and 946.7% increase) 32, 33. As self-complementary AAV produces gene expression more rapidly than AAV and higher levels of transduction 43, the difference in survival between AAV8 and scAAV8 may once again demonstrate that early up-regulation of SMN protein levels is critical. Although scAAV9 intravenous injection does give robust SMN expression and a significant increase in survival, in a head-to-head comparison with direct ICV injection into the CSF, the latter gives significant increase in weight gain (∼10% increase for intravenous injection and ∼110% increase for ICV injection) and lifespan over the former (15% increase in survival for intravenous injection compared to 31% for ICV injection) 44, demonstrating the dramatic difference that the route of administration can make.

Interestingly in the perspective of clinical trials, non-human primate studies have shown that scAAV9 can still enter the CNS following IV injection transducing motor neurons 32. The major current limitation of this technology is the large-scale production sufficient for older children or adults. However, enough viruses could be produced for a clinical trial targeting young children or CSF delivery and an SMA clinical trial is planned within the next 1 year (http://www.fsma.org/LatestNews/index.cfm?ID=7444).

Another major point that needs to be considered in the prospective of translating this technology to clinical trials is the way of delivery, to optimize the transduction of motoneurons, to reduce toxicity and to be less invasive as possible.

However, gene therapy gave very promising results in pre-clinical studies and hence a translation in humans seems to be feasible.

## Stem-cell therapy

Another treatment strategy that is being actively investigated in SMA is cell transplantation for neuroprotection and ultimately cell replacement (Table 1, Fig. 1C). Embryonic stem cells can be differentiated into neural stem cells and then functional motor neurons by using retinoic acid, sonic hedgehog and neurotrophic factors 45, 46. We previously demonstrated that there are some *in vivo* benefits of neural stem-cell intrathecal injections in severe SMA mice after differentiating neural stem cells from mouse spinal cord neurospheres 47, 48. Spinal muscular atrophy-treated mice displayed increased survival and motor behavioural benefits, as well as increased motor neurons number and size 48. A work by California Stem Cell Inc. (Irvine, CA, USA) has recently resulted in the development of new methods to create high purity human motor neuronal progenitor cells 49. This group has also demonstrated that transplantation of these cells leads to modest neuropathological and phenotypic benefits in an SMA mice model 50. They proposed a clinical trial in SMA patients based on intraspinal injections of their cells, but until now, FDA put on hold this clinical study.

Our group 51 investigated the feasibility of genetically engineered induced pluripotent stem (iPS) cells to generate cells to be used as disease model and as cell source for cell transplantation. We generated human SMA iPSCs by using non-viral, non-integrating episomal vectors and then performed genome editing with oligonucleotides to modify *SMN2* to produce a functional *SMN1*-like protein. We generated iPSC lines from two type I SMA patients by using a non-viral vector method based on nucleofection of adult fibroblasts with reprogramming factors. Using single-stranded DNA oligonucleotides into the cells, we induced a genome editing of *SMN2* promoting the exchange of a T to C at position +6 of exon 7, thus modifying the coding region of *SMN2* to a more *SMN1*-like sequence. Uncorrected SMA-iPSC-derived motor neurons reproduced disease-specific features, which were ameliorated in motor neurons derived from genetically corrected SMA-iPSCs. Upon direct transplantation into a severe SMA mouse model, wild-type and corrected SMA-iPSC-derived motor neurons engrafted in the spinal cord. Moreover, the transplantation led to an improvement in the disease phenotype, ameliorating muscle connections, the physical appearance of the mice with an increase in bw (∼50%) and slightly extending also the lifespan (∼40% increase). Furthermore, all of the transplanted human motor neurons both *in vitro* and *in vivo* release growth factors, which could be at the basis of transplantation beneficial effects. Overall, our study demonstrates the feasibility of generating patient-specific iPSCs and their motor neuron progeny that are genetically corrected and free of exogenous, and suggests the potential of this approach for clinical translation 51.

Although promising, it seems obvious that the use of a therapeutic approach based on stem cells leads to results that are not comparable at all to the ones obtained with gene and/or molecular therapy in terms of improvement in the phenotype of the models and survival. As a matter of fact, our group found an increase in lifespan of ∼40% using cellular therapy, whereas molecular therapy and gene therapy were shown to increase survival of more than 1500%, depending on the viral vector and type of ASOs used. Therefore, our belief is that the main prospect of non-SMN targeted therapies, such as stem cells, could be their use in patients who are already symptomatic with the aim of improving the phenotype and preventing an additional damage of motor neurons, exploiting the ability of stem cells to produce neurothropic and growth factors. Overall, our conclusion is that stem cells and molecular or gene therapy could be combined to reach the best therapeutic effect. As a matter of fact, we suggest that the ideal therapeutic approach for SMA will be resolving the genetic defect with gene therapy or ASOs as well as improving the signs of the disease with other strategies like stem-cell transplantation. However, before any applications to humans, safety and efficacy of cellular therapy need to be carefully proved in a pre-clinical setting.

## Conclusions

Currently, no effective treatment is available for motor neuron disorders such as SMA and the main therapeutic strategies that are now in use are based on symptomatic treatments and supportive care through the use of orally administered drugs. At a preclinical level, many therapeutic strategies have been investigated and, in this review, we presented the most interesting ones. As regards molecular therapy, the use of ASOs to redirect *SMN2* gene splicing and improve exon 7 inclusion in the majority of the transcript seems to be the most promising approach. In particular, the use of an ASO of 25 nucleotides with Morpholino chemistry seems to have the highest therapeutic potential. Nevertheless, an important issue to discuss is whether a therapeutic window for SMN increase exists. Le *et al*. highlight that early induction of SMN at post natal days 3 and 4 leads to the highest effect on survival 52. Later induction (post natal day 6), even though still promotes some rescue, has a much diminished response. Therefore, this observation suggests that the best strategy to treat SMA involves an induction of SMN at the earliest stages of the disease course. Also, Lutz *et al*. focused their attention on this topic and their findings suggest that there is a therapeutic window from post natal day 4 through post natal day 8 defined by a highest improvement in neuromuscular synapse pathology and by the ability of motor neurons to respond to SMN induction, following which restoration of SMN protein failed to produce therapeutic benefits 53. Overall, it will be fundamental to take into consideration the therapeutic window issue when planning human clinical trials for SMA. Gene therapy seems to be most efficient in improving survival in a severe mouse model of SMA, although a better definition of the route of administration and of the safety profile of the viral vectors used is needed to allow an efficacious translation of this approach to the clinics. As regards stem-cell therapy, a thorough understanding of motor neuron disorders specific pathways must grow in parallel with progresses in understanding stem-cell biology, with the aim of obtaining reliable experimental information and adequate cell sources.

Clinical trials are ongoing with antisense oligonucelotides and are expected soon for gene therapy. One of the crucial points in successful clinical translation of molecular and even cellular approaches will be the appropriate design of clinical trials that include the identification of the appropriate patient populations that can demonstrate the efficacy of a specific treatment.
